# Knowledge, attitudes, and practices regarding vaccination among community pharmacists

**DOI:** 10.1017/S1463423622000330

**Published:** 2022-07-22

**Authors:** Nesligul Ozdemir, Emre Kara, Aygin Bayraktar-Ekincioglu, Ayse Buyukcam, Ayce Celiker, Kutay Demirkan, Ates Kara

**Affiliations:** 1 Hacettepe University Faculty of Pharmacy, Department of Clinical Pharmacy, Ankara, Turkey; 2 Hacettepe University, Faculty of Medicine, Department of Pediatric Infectious Diseases, Ankara, Turkey

**Keywords:** attitude, behavior, community pharmacist, influenza vaccine, vaccination rate

## Abstract

**Background:**

Healthcare professionals’ vaccine recommendation is the most effective method to increase vaccination rates of the community. The vaccine counseling and recommendation behavior of pharmacists, who are among the easily accessible healthcare professionals, are influenced by their knowledge and attitudes about vaccines.

**Aim:**

It was aimed to investigate community pharmacists’ knowledge, attitudes, and practices regarding commonly used vaccines.

**Methods:**

A cross-sectional study was conducted as an online survey with a sample of 1100 community pharmacists in Turkey. Pharmacists were invited to participate in the study by phone calls. A structured survey, which consists of 40 questions to assess the knowledge, attitudes, and practices regarding vaccines, was sent to the e-mail addresses of pharmacists who volunteered to participate in the study.

**Findings:**

A total of 430 pharmacists completed the survey. Thirty percent of pharmacists had lack of knowledge about vaccination during pregnancy, whereas 52.2% and 31.4% of pharmacists believed that tetanus and influenza vaccines should be provided during pregnancy, respectively. Nearly 89% of pharmacists recommended vaccines to patients, mainly for influenza vaccine (83.9%). Only 31.5% of pharmacists had been vaccinated against influenza in the last season, whereas 50.5% had never been vaccinated. Pharmacists who had been vaccinated with influenza vaccine had a high rate of recommending influenza vaccines to the patients.

**Conclusion:**

The present study found that vaccination among pharmacists in Turkey and their knowledge on vaccination during pregnancy were low. Further education of pharmacists to improve their knowledge and attitudes toward vaccines is needed.

## Introduction

Effective immunization in the community can be achieved by increasing vaccination rates both in adults and pediatrics. Although vaccination rates have been increased worldwide, some countries have not reached adequate rates in both child and adult populations (Williams *et al.*, 2017; Centers for Disease Prevention and Control, 2017; World Health Organization, 2020). Despite the achievement of the desired goals for childhood vaccination, adult immunization is behind the desired target in Turkey (Ozisik *et al.*, 2016; World Health Organization, 2019).

Biologic, socioeconomic, epidemiologic, and logistic features are reported to be the main factors that affect vaccination rates (World Health Organization, 2020; Glatman et al., 2012). Furthermore, misperception and lack of knowledge on vaccines leads to prejudices that result in vaccine hesitancy and anti-vaccination movement, which generate an important health issue (Kata, 2010; Isaacs, 2019).

The decision of the community on being vaccinated is mutually influenced by recommendations of healthcare professionals (HCPs) and vaccination-related beliefs and behaviors of HCPs (Collange *et al.*, 2016; MacDougall et al., 2015). Lack of knowledge and negative attitudes of HCPs on vaccines can diminish the trust of the community in the importance of vaccination, and subsequently trigger antivaccination movements (European Centre for Disease Prevention and Control, 2015; Paterson *et al.*, 2016; Barrett *et al.*, 2018). Given the fact that family physicians, nurses, and pharmacists are responsible for providing preventive health services, their professional attitudes and beliefs on vaccination can constitute a sanction power over the community.

It has been known that community pharmacists are easily accessible HCPs and can play an active role in vaccination by counseling, supplying vaccines, and performing vaccination to enhance vaccination coverage of the community (Isenor *et al.*, 2016; American Public Health Association, 2019; Ciliberti *et al.*, 2020). On the other hand, pharmacists’ knowledge of vaccines may affect their attitudes toward vaccine advice, which reflects the need for raising awareness of vaccination among HCPs (Ciliberti *et al.*, 2020).

It is essential to reveal the factors that affect HCPs’ vaccination-related attitudes and behaviors to provide effective and appropriate preventive healthcare services. Although studies have evaluated the impact of the pharmacist as a vaccine practitioner, a limited number of studies were focused on pharmacist’s knowledge, attitudes, and behavior about vaccines and vaccination (Barrett *et al.*, 2018; Ciliberti *et al.*, 2020; Scarpitta *et al.*, 2019; Della Polla *et al.*, 2020; Valiquette et al., 2015; Toledo *et al.*, 2017; Dolan *et al.*, 2012; Tolentino *et al.*, 2018). In particular, not many studies were undertaken in developing countries, where community pharmacies are the most preferred source of health information, therefore raising awareness of pharmacists becomes crucial. This study was aimed to determine the knowledge, attitudes, and practices of community pharmacists about vaccines and to identify potential barriers and related factors on vaccination advice.

## Methods

### Study design

This descriptive, cross-sectional study was conducted as an online survey among community pharmacists between July 2017 and March 2018 in Turkey. A structured survey was designed following the literature review by two infectious diseases specialists and three clinical pharmacists.

A pilot study was conducted to test and validate the understandability of the questions among ten pharmacists, and the questionnaire was finalized with minor revisions. The survey consisted of 40 questions organized into four sections (demographics; knowledge, attitudes, and behaviors on vaccination in general; knowledge of vaccination during pregnancy; and perceived knowledge, attitudes, and behaviors on influenza vaccine), which takes 15–20 minutes to complete. In the questionnaire, ‘vaccines’ refers to the vaccines recommended by the Ministry of Health for all age groups in the population including the ones for healthcare workers and pregnancy. The perceived knowledge was assessed by using a 5-point Likert scale (very low (1), below average (2), average (3), above average (4), very high (5)) and categorized as average and below average (1-3) and above average (4-5) for the analysis.

### Participants

In Turkey, community pharmacists are the owner of pharmacies (no chain pharmacies), and the total number of pharmacies was reported as 25,453 in 12 statistical regions (Turkey as a candidate country of the European Union uses the Nomenclature of Territorial Units for Statistics (NUTS) determined by Eurostat) in the year of 2017 (NUTS Maps, 2020; Türk Eczacıları Birliği, 2017). Community pharmacists who were practicing in Turkey during the study period and willing to participate were considered eligible for participation. A representative sample was considered for the study in the view of numbers and distribution of pharmacies in Turkey, therefore it was aimed to achieve a total of 1100 participants by targeting at least one pharmacist in each province. The list containing telephone numbers of the pharmacies in each province was created following Google^®^ search, and pharmacies were randomly chosen from this list. The pharmacists were invited to the study by clinical pharmacists via phone calls. Each pharmacy was called 3 times at most (on the same day) and removed from the list if there was no answer. The survey link (as Google^®^ Forms) was sent via e-mail, if pharmacists agreed to participate.

The main outcome measures were pharmacists’ knowledge about influenza vaccine and vaccination during pregnancy, vaccine recommendation to the patients, and pharmacists’ vaccination status. Determining the factors affecting the vaccine recommendation behavior was defined as a secondary outcome.

### Statistical analysis

IBM SPSS Statistics for MacOS, version 23.0 (IBM Corp., Armonk, N.Y., USA) was used for the analysis. Mean ± standard deviation (SD), median (minimum-maximum), and frequency were used as descriptive statistics. Chi-square test, Fisher Exact test, or McNemar test were used for categorical variables, and Student-t-test, Mann-Whitney U test, or Kruskal-Wallis test was used for continuous variables, where appropriate. Univariable and multivariable logistic regression models were used to identify risk factors associated with vaccination recommendations. Independent variables that were found to be significant predictors (p < 0.05) were further included in the logistic regression models. A *P*-value <0.05 with a 95% confidence Interval was considered significant.

## Results

A total of 430 (39.1%; *n* = 1100) community pharmacists participated in the study; however, 2 sets of responses were excluded due to missing data, which lead to 428 answers (*n* = 297; 69.4% female) for the analysis. The median age of pharmacists was 41 (20–74) years, and experience in profession was 15 (0.25–49) years. A minority of participants (*n* = 90, 21.0%) had a postgraduate degree (master or doctorate), and 75.9% (*n* = 325) of pharmacies were located near to hospital/health center or on the main street.

**Figure 1.**
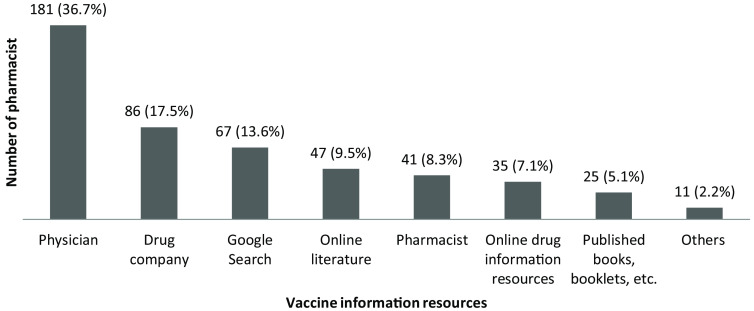
Sources of information on vaccines stated by pharmacists. *Others (n): academician (5), nurse (4), midwife (2).

Two hundred eighty-five pharmacists (66.6%) stated that they previously received information and/or training about vaccines, and main sources of information were indicated as undergraduate education (*n* = 145, 33.9%), drug companies (*n* = 128, 29.9%), continuing professional education courses (*n* = 121, 28.3%), other related seminars and courses (*n* = 35, 8.2%), medical literature (*n* = 26, 6.1%), or others (*n* = 7, 1.6%). Furthermore, pharmacists (36.7%) indicated that they gather information about the vaccines from physicians if required, while 41 (9.6%) stated that they did not acquire any information (Figure 1).

Female (22.9% versus 13.7%, *P* = 0.029), older (median 48 versus 39 years, *P* < 0.001), and experienced (median 23 versus 13 years, *P* < 0.001) pharmacists were more likely to seek information from drug companies. However, younger (median 35 versus 42 years, p = 0.018) and less experienced (median 11 versus 15 years, *P* = 0.026) pharmacists preferred to use online drug information resources for vaccine information. Having information from the literature (9.5% versus 15.7%, *P* = 0.052) and books/booklets (4.4% versus 11.1%, *P* = 0.016) were more likely preferred or used by pharmacists with a postgraduate degree.

### Knowledge about vaccines and vaccination

**Figure 2.**
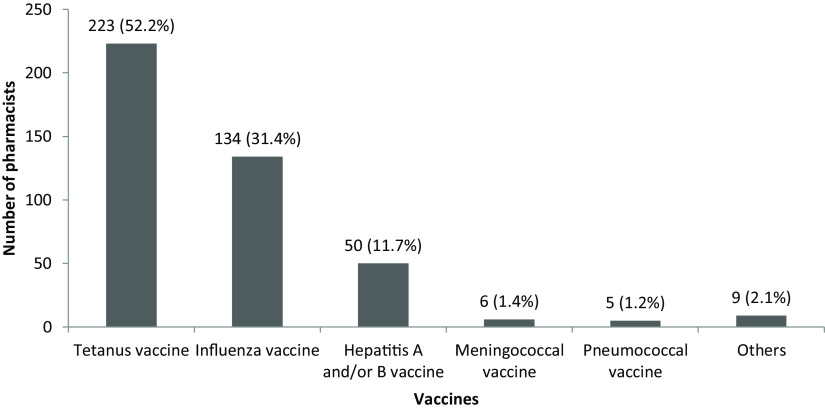
Pharmacists’ knowledge of vaccination during pregnancy. *Others (n): measles-mumps-rubella (4), rabies (2), varicella (1), rubella (1), and pertussis (1).

Regarding vaccination during pregnancy, 294 (68.7%) pharmacists stated that ‘there are vaccines that pregnant women should have’, whereas the rest of them stated either ‘do not know’ (25.9%) or ‘no vaccine that pregnant women should have’ (5.4%). In addition, 128 (29.9%) pharmacists indicated that not have any knowledge about vaccination during pregnancy, whereas 52.2% and 31.4% of pharmacists believed that tetanus and influenza vaccines should be done during pregnancy, respectively (Figure 2). Being female (odds ratio 3.098 (2.005-4.785), 95% CI, *P* = 0.000) and having above average level of knowledge about vaccines [odds ratio 1.810 (1.149-2.852), 95% CI, *P* = 0.011] were related to knowing that vaccinations should be done in pregnant women.

**Figure 3.**
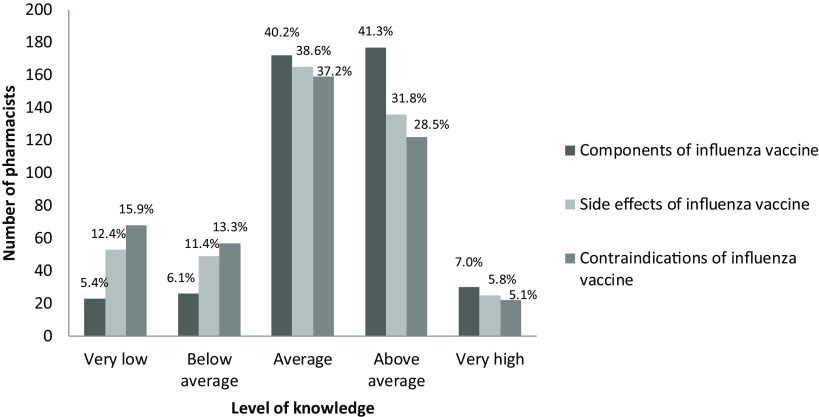
The level of pharmacists’ perceived knowledge about components and safety of the influenza vaccine.

Pharmacists were asked to indicate their perceived level of knowledge on components and safety (side effects and contraindications) of the influenza vaccine (Figure 3); the level of knowledge on contraindications increased with age (median 45 versus 40 years, *P* = 0.028). The factors that differ in terms of pharmacists’ knowledge about the influenza vaccine are given in Table 1.

**Table 1.** Parameters that differ in terms of knowledge and behavior of pharmacists about influenza and diphtheria/tetanus vaccine (*n*=428)

	Gender			Education			Years in profession				
	Male (*n*=131)	Female (*n*=297)	*P* ^*^	Bachelor (n=338)	Postgraduate (*n*=90)	*P* ^*^	0–10 (*n*=152)	11–20 (*n*=124)	21–30 (*n*=90)	>30 (*n*=62)	*P* ^*^
Pharmacist’s perceived knowledge about vaccines, % (*n*)											
Are there any vaccination recommendations during pregnancy?											
Yes	51.2 (67)	76.4 (227)	<0.001	67.2 (227)	74.4 (67)	0.228	58.6 (89)	79.0 (98)	76.7 (69)	61.3 (38)	<0.001
No	9.9 (13)	3.4 (10)		6.2 (21)	2.2 (2)		5.3 (8)	0.8 (1)	7.8 (7)	11.3 (7)	
Don’t know	38.9 (51)	20.2 (60)		26.6 (90)	23.3 (21)		36.2 (55)	20.2 (25)	15.6 (14)	27.4 (17)	
Level of knowledge about components of influenza vaccine											
Above average	39.7 (52)	52.2 (155)	0.017	45.6 (154)	58.9 (53)	0.025	42.8 (65)	48.4 (60)	52.2 (47)	56.5 (35)	0.254
Average and below average	60.3 (79)	47.8 (142)		54.4 (184)	41.1 (37)		57.2 (87)	51.6 (64)	47.8 (43)	43.5 (27)	
Level of knowledge about side/adverse effects of influenza vaccine											
Above average	34.4 (45)	39.1 (116)	0.354	34.9 (118)	47.8 (43)	0.025	30.9 (47)	37.9 (47)	48.9 (44)	37.1 (23)	0.051
Average and below average	65.6 (86)	60.9 (181)		65.1 (220)	52.2 (47)		69.1 (105)	62.1 (77)	51.1 (46)	62.9 (30)	
Level of knowledge about contraindications of influenza vaccine											
Above average	68.7 (90)	65.3 (194)	0.495	30.2 (102)	46.7 (42)	0.003	25.7 (39)	37.9 (47)	46.7 (42)	25.8 (16)	0.003
Average and below average	31.3 (41)	34.7 (103)		69.8 (206)	53.3 (48)		74.3 (113)	62.1 (77)	53.3 (48)	74.2 (46)	
Pharmacist’s vaccination status											
Being vaccinated with diphtheria/tetanus vaccine											
Vaccinated	26.0 (34)	44.4 (132)	<0.001	35.2 (119)	52.2 (47)	0.003	44.1 (67)	42.7 (53)	41.1 (37)	14.5 (8)	<0.001
Not vaccinated	74.0 (97)	55.6 (165)		64.8 (219)	47.8 (43)		55.9 (85)	57.3 (71)	58.9 (53)	85.5 (53)	
Being vaccinated with influenza vaccine											
Vaccinated	33.6 (44)	30.6 (91)	0.545	30.5 (103)	35.6 (32)	0.357	27.0 (41)	31.5 (39)	33.3 (30)	40.3 (25)	0.282
Not vaccinated	66.4 (87)	69.4 (206)		69.5 (235)	64.4 (58)		73.0 (111)	68.5 (85)	66.7 (60)	59.7 (37)	

* Chi-square test, Fisher Exact test, or McNemar test was used.

### Vaccination status of pharmacists

Two hundred twenty-four pharmacists (52.3%) stated that they had not been vaccinated against diphtheria and tetanus in the past 10 years, while 38 (8.9%) did not remember whether they were vaccinated or not. One hundred thirty-five (31.5%) pharmacists had been vaccinated against influenza in the last season and 216 (50.5%) had not been vaccinated at all. Almost half of the pharmacists (*n* = 209, 48.8%) had not received any influenza vaccines, and only 52 (12.1%) pharmacists indicated to have vaccines every year during the last 5 years. Pharmacists’ influenza vaccination behavior was not statistically different in terms of gender, education level, and years in the profession (*P* > 0.05) (Table 1). It was found that the rate of having influenza vaccine is high in pharmacists with high perceived level of knowledge on contraindications of vaccine (>average = 38.2% versus ≤average = 28.2%, *P* = 0.003), but it did not show difference in terms of the level of knowledge on side effects and components of the vaccine. However, having above average level of general knowledge about vaccines (odds ratio 1.926 (1.262-2.938), 95% CI, *P* = 0.002), in particular with the knowledge about the side effects (odds ratio 1.741 (1.148-2.640), 95%CI, *P* = 0.009), content (odds ratio 1.818 (1.203-2.748), 95%CI, *P* = 0.005), and contraindications (odds ratio 1.576 (1.031-2.408), 95%CI, *P* = 0.036) of vaccines were found to be positive determinants of being vaccinated with influenza vaccine.

Vaccination rates with diphtheria vaccine were higher among young pharmacists (39 versus 44.5 years, *P* = 0.001). The other factors that differ in terms of vaccination status with diphtheria vaccine were given in Table 1. Being female (odds ratio 2.282, 1.451-3.589, 95% CI, *P* = 0.000), having postgraduate education (odds ratio 2.012, 1.257-3.218, 95% CI, *P* = 0.004), and being vaccinated with influenza vaccine (odds ratio 2.019, 1.333-3.060, 95% CI, *P* = 0.001) increase the vaccination rates for diphtheria and tetanus by twofold.

### Pharmacists’ advice on vaccination

**Figure 4.**
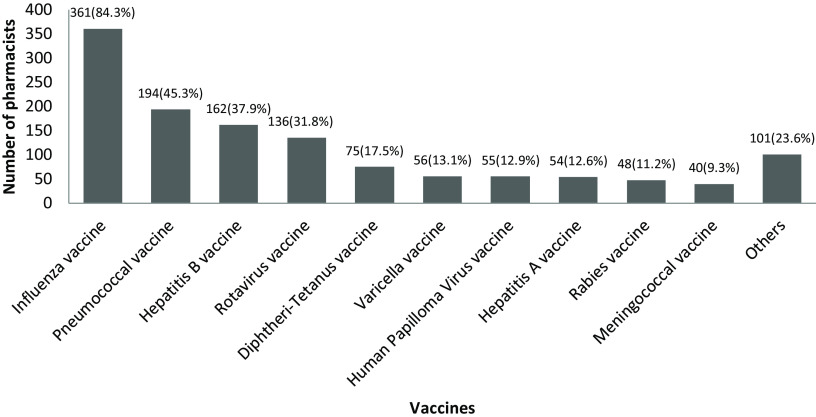
The vaccines are recommended by pharmacists to patients. *Others (n; %): Bacillus Calmette-Guérin vaccine (28; 6.5%), polio vaccine (24; 5.6%), tetanus-diphtheria-pertussis vaccine (23; 5.4%), measles-mumps-rubella vaccine (14; 3.3%), typhoid vaccine (7; 1.6%), Zoster vaccine (5; 1.2%).

It was shown that 379 (88.6%) pharmacists recommended vaccines to patients, mainly for influenza vaccine (Figure 4).

A majority of pharmacists stated that they recommend vaccines to individuals ≥65 years (82%) and with chronic diseases (75.2%) in general. However, they recommend vaccines relatively less to children (48.1%) and pregnant women (12.1%). The influenza vaccine was recommended to patients by 83.9% of pharmacists, but only 57.2% of them recommended to their staff at the pharmacy.

In particular, influenza vaccine was recommended by pharmacists to people aged ≥65 years (78.5%) and 2–64 years with the risk factors (71.5%), but less likely recommended for children under 2 years (4.2%) and pregnant women (7.9%).

It was found that the pharmacist’s level of knowledge about influenza vaccination, the content of the vaccines, the side effects of the vaccines, and the contraindications of the vaccines showed a statistically significant difference in terms of pharmacists recommending vaccines to patients and pharmacy staff (*P* < 0.05).

Pharmacists vaccinated with influenza vaccine were more likely to recommend influenza vaccine to the patients (95.6% versus 78.5%, *P* < 0.001) and pharmacy staff (93.3% versus 40.6%, *P* < 0.001). Furthermore, the recommendation rates for pharmacy staff (66% versus 52.8%, *P* = 0.009) and patients (91% versus 80.3%, *P* = 0.004) were higher among pharmacists who have above level of knowledge on contraindications of the influenza vaccine.

The odds ratios of the factors affecting the behavior of recommending vaccines to patients or pharmacy staff are shown in Table 2.

**Table 2.** Univariate and multivariate analysis of risk factors influencing pharmacists’ behavior in recommending vaccines to their patients and staff

Parameters	Univariate analysis				Multivariate analysis			
	Recommending any vaccines to patients		Recommending any vaccines to employees		Recommending any vaccines to patients		Recommending any vaccines to employees	
	OR (lower-upper value) 95.0% CI	*P* value	OR (lower-upper value) 95.0% CI	*P* value	OR (lower-upper value) 95.0% CI	*P* value	OR (lower-upper value) 95.0% CI	*P* value
Having above average level of knowledge about vaccines	2.516 (1.185-5.342)	0.016	1.875 (1.236-2.845)	0.003				
Having above average level of knowledge about the content of the seasonal influenza vaccine	2.327 (1.227-4.414)	0.010	1.892 (1.282-2.792)	0.001				
Having above average level of knowledge about the side effects of the seasonal influenza vaccine	3.470 (1.583-7.606)	0.002	1.852 (1.234-2.779)	0.003				
Having above average level of knowledge about the contraindications of the seasonal influenza vaccine	5.097 (1.974-13.156)	0.001	1.732 (1.142-2.626)	0.010	3.980 (1.060-14.941)	0.041		
Being vaccinated with influenza vaccine	5.943 (2.091-16.855)	0.001	20.471 (10.011-41.859)	<0.001	5.300 (1.841-15.259)	0.002	19.305 (9.415-39.586)	<0.001

In addition, gender was found to be a significant factor for pharmacists recommending influenza vaccine in different age groups; female pharmacists mostly recommend the influenza vaccine to people aged <65 years (45.8% versus 30% in males) and male pharmacists mostly recommend to people over the age of 65 (70% versus 54.2% in females) (*P* = 0.005).

There was no statistical difference in terms of education level, professional experience, and gender in the behavior of pharmacists recommending general vaccinations to the patients and influenza vaccination to patients and staff (*P* > 0.05).

Ninety percent of pharmacists (*n* = 386) indicated that vaccine administration should be legal in the pharmacies, 40% of those believed that this regulation will allow convenience to patients and may increase the vaccination rates.

## Discussion

In this study, pharmacists’ opinions on vaccines and attitudes towards vaccination were examined. It was found that pharmacists’ knowledge about vaccination during pregnancy was low and that less vaccine recommendations were made to pregnant women and children. While nearly 90% of pharmacists stated that they generally recommend vaccines to patients, influenza vaccine comes first among the recommended vaccines. However, vaccines such as pneumococcal, hepatitis b, and diphtheria-tetanus were recommended by a small proportion of pharmacists. The proportion of pharmacists who got influenza and diphtheria-tetanus vaccine was found to be quite low.

It is essential for pharmacists to access and use up-to-date vaccine-related information for counseling with the public. The sources used by pharmacists as a reference for vaccines are varied; literature, social media, internet, and education/training activities are among the most preferred resources (Della Polla *et al.*, 2020). It was observed in this study that nearly 35% of pharmacists did not receive any training or information on vaccines, and 40% did not use any source of information about the vaccine. Remarkably physicians had been the most preferred information source for pharmacists followed by drug companies and the internet. In the study of Della Polla et al., while the proportion of pharmacists (40%) who received training/information on vaccines were similar to the present study, the most preferred sources of information (literature, internet and educational activities) were different; besides, gender, age, education level, and years of professional experience affected the preferences of pharmacists on information sources about vaccines (Della Polla *et al.*, 2020). In this study, it was seen that pharmacists mainly consulted physicians or drug company representatives about the subjects they did not know about vaccines, and they preferred to look at the literature less. The demand for a faster response due to pharmacists’ workload may have caused this situation.

Tetanus and influenza vaccines are recommended to all pregnant women, and pregnant women can have other vaccines, except live vaccines, according to the presence of other risk factors (Centers for Disease Prevention and Control, 2018). The pharmacists’ knowledge about vaccination during pregnancy was low in this study. One in six pharmacists has misinformation about routine vaccinations during pregnancy. The rates of healthcare workers having the correct information about tetanus and influenza vaccination in pregnant women are between 60 and 70% (Barrett *et al.*, 2018; Dube et al., 2020). In this study, knowing the vaccinations that should be done in pregnant women was associated with the gender and the level of knowledge about vaccines of the pharmacist. Compared to other healthcare professionals, the rate of pharmacists who know the necessity of tetanus and influenza vaccines in pregnant women was quite low in this study. The fact that pharmacists encounter fewer pregnant women compared to women with chronic diseases may be one of the reasons why they have less up-to-date information about vaccination during pregnancy.

Only 12% of pharmacists recommended vaccines to pregnant women in daily practice, and the proportion was even lower (7.4%) for influenza vaccine. The rates of pharmacists recommending influenza vaccines to pregnant women varies between 15% and 71% (Barrett *et al.*, 2018, Dube et al., 2020; Dube et al., 2019). Vaccination for pregnant women is recommended within adult vaccination program, but vaccination status is not routinely followed up by family physicians. These patients can easily contact community pharmacist and be followed up about vaccination status. Pharmacists are required to increase their knowledge on routine vaccination program for pregnant women in order to play an active role.

The recommendation of influenza vaccine for patients aged under and over 65 years differed; male pharmacists recommend influenza vaccines for patients over 65 years, whereas female pharmacists tended to recommend for patients under 65 years of age and with the risk factors such as chronic diseases. These differences in the attitudes of pharmacists may lead them to ignore the remaining patients. The rate of recommending influenza vaccine to patients was higher among pharmacists with a high level of knowledge about contraindications of influenza vaccine. This shows that knowing the vaccine’s safety requirements enables pharmacists to recommend vaccines to their patients more confidently.

Pharmacists’ vaccination status had a significant effect on recommending influenza vaccine to patients in the present study. Pharmacists who vaccinated against influenza were mostly recommending influenza vaccine to both patients and pharmacy staff. It was known that vaccinated HCPs more likely to recommend vaccines to patients (Scarpitta *et al.*, 2019; Della Polla *et al.*, 2020; Valiquette et al., 2015). On the contrary, situations such as the pharmacists’ lack of confidence in the vaccine, being unvaccinated, having less professional experience, and not knowing the consequences of the disease reduce the likelihood of recommendation of vaccines by pharmacists (Barrett *et al.,* 2018; Toledo *et al.*, 2017; Dolan *et al.*, 2012). Unlike the literature, year of experience and educational level did not have a significant effect on the influenza vaccine recommendation to patients in this study.

The number of pharmacists who recommend influenza vaccine to patients was high compared to recommending the vaccine to pharmacy staff. The priority in provision of healthcare services may have caused vaccine recommendations to pharmacy staff to be remained at the background. This indicates the need to increase awareness of pharmacists on the vaccination status of the pharmacy staff, who is an assistant healthcare service provider.

The healthcare workers who have face-to-face contact with patients should protect both themselves and patients against infectious diseases. Among healthcare professionals, especially physicians and healthcare workers working in hospital settings had higher vaccination rates (Ciftci *et al.*, 2018). Influenza vaccination rates of pharmacists were found to be lower than physicians (98%) and nurses (92%) (Centers for Disease Prevention and Control, 2020). The vaccination rates of pharmacists with influenza vaccine (86% versus 51%) in the past five years and tetanus vaccine (65% versus 39%) in the last 10 years were low in this study compared to the study of Valiquette *et al.* (Valiquette et al., 2015). The fact that pharmacists have shorter contact with patients compared to physicians and nurses which may have caused to feel safer and less aware of protection from infectious diseases.

In the present study, the factors affecting the attitudes of pharmacists regarding vaccination with tetanus vaccine were determined as female gender, younger age, higher education level, and having fewer years of experience. However, these characteristics did not affect influenza vaccination status. On the other hand, pharmacists who had more knowledge about the safety of the influenza vaccine were more likely to be vaccinated.

In regard with pharmacists’ perceived level of knowledge about the components and safety of influenza vaccine, age and year of experience were found to be not affecting factors in this study. On the other hand, being female and having high level of education have an impact on the knowledge about components and safety of the vaccine. This finding shows that pharmacists need to update their knowledge on vaccination in order to provide adequate healthcare services.

One of the potential areas where pharmacists can contribute to vaccination is working as a vaccine practitioner. Pharmacists are authorized by regulatory issues to administer certain vaccines in the United States of America, Canada, Australia, and some European countries (International Pharmaceutical Federation, 2016). Studies have shown that allowing pharmacists to vaccinate in the pharmacy increases the rate of the recommendation of vaccines and vaccination status of patients (International Pharmaceutical Federation, 2016). Community pharmacists in Turkey do not have an authority to administer the vaccine in pharmacies. In this study, most of the pharmacists wanted to have an authority to vaccinate. Pharmacists believed that this new role will make it easier for patients to access vaccines, thereby increase vaccination rates.

### Limitations

This study had some inevitable limitations. Although the number of participants is high, it may not be sufficient to reflect pharmacists’ beliefs and attitudes across the country. Participants were not selected according to regions with different socioeconomic levels, and therefore the effect of this situation on the vaccine attitudes and behaviors of pharmacists could not be examined. In areas where the perceived level of knowledge is evaluated, these levels are entirely dependent on the statement of the participant and may not reflect the true level of knowledge.

## Conclusion

In this study, the rate of pharmacists having been vaccinated was low compared to the literature. The behavior of recommending vaccines to patients and pharmacy staff was positively affected by the pharmacists’ own vaccination status and its high level of knowledge about vaccine contraindications. This study provided an insight on the pharmacists’ professional attitudes and knowledge on vaccines and identified requirements on the vaccination practice. This study demonstrates the need to educate pharmacists to improve their beliefs and attitudes toward vaccines in order to increase the rate of vaccination in the community.
